# Pharmacokinetic study of *Strongylocentrotus nudus* egg polysaccharide in rats and beagles using a ^3^H-labeling method

**DOI:** 10.3389/fphar.2023.1109084

**Published:** 2023-03-02

**Authors:** Han Xing, Xiaojie Zhu, Jianmin Liao, Ying Kong, Yayuan Lu, Di Zhao, Ning Li, Xijing Chen, Zhiying Qin

**Affiliations:** ^1^ Department of Pharmacy, First Affiliated Hospital of Zhengzhou University, Zhengzhou, China; ^2^ Henan Key Laboratory of Precision Clinical Pharmacy, Zhengzhou University, Zhengzhou, China; ^3^ Henan Engineering Research Center for Application and Translation of Precision Clinical Pharmacy, Zhengzhou University, Zhengzhou, China; ^4^ Clinical Pharmacokinetics Laboratory, China Pharmaceutical University, Nanjing, Jiangsu Province, China

**Keywords:** *Strongylocentrotus nudus* egg polysaccharide, ^3^H-labeling method, pharmacokinetics, tritium, beagles, rats

## Abstract

*Strongylocentrotus nudus* egg polysaccharide (SEP) extracted from sea urchins has potential anticancer activity. However, little is known about its pharmacokinetic properties. To investigate the pharmacokinetics of SEP, it was radiolabeled with tritium. Furthermore, a sensitive, selective, and rapid liquid scintillation counter (LSC) method for quantifying ^3^H-SEP in biological matrix was validated. The lower quantification limit of the method was 4 Bq. The relative standard deviations (RSDs) of the intra- and inter-day precision were <3.0% and <3.9%, respectively. ^3^H-SEP was successfully applied to investigate the pharmacokinetics of SEP after intravenous administration of 20, 40, and 80 mg/kg (40 μCi/kg) in rats and 5, 10, and 20 mg/kg (6 μCi/kg) in beagles. The AUC_(0-t)_ of SEP at three different doses was 487.81 ± 39.99 mg/L*h, 1,003.10 ± 95.94 mg/L*h, and 2,188.84 ± 137.73 mg/L*h in rats and 144.12 ± 3.78 mg/L*h, 322.62 ± 28.03 mg/L*h, and 754.17 ± 37.79 mg/L*h in beagles. The terminal elimination half-life (t_1/2_) of SEP was longer in beagles (204.29 ± 139.34 h) than in rats (35.48 ± 6.04 h). The concentration of SEP in plasma declined rapidly in both rats and beagles. All the study results provide detailed pharmacokinetic profiles of SEP in two kinds of animals, which will be helpful for further development.

## 1 Introduction

Cancer treatment is still a problem around the world. We are continuously seeking an antineoplastic therapy with minor side effects. Compared with other treatments like surgery and radiotherapy, the use of cancer drugs—including chemotherapy and biologics—is currently the most effective treatment for metastatic cancers (Behranvand et al., 2022). The development of anticancer compounds brings new hope to cancer treatment. Unfortunately, even patients treated with advanced therapies and techniques can survive for only a few months due to the severe toxicity and side effects caused by anticancer compounds (Zraik and Heß-Busch, 2021). For example, doxorubicin and paclitaxel exert eminent effects on liver, breast, ovarian, and lung cancer. However, their application is limited due to their acute and chronic adverse effects. The most representative side effect is the dose-limiting myelosuppression and cardiac toxicity caused by doxorubicin; nausea and vomiting are also very common (Li et al., 2018; Zhu and Lin, 2021). Thus, side effects have also been considered in screening new anticancer compounds. Compared with chemotherapy drugs, biological macromolecules, especially natural active compounds including polysaccharides, may have a bright future in the treatment of human malignancies with fewer side effects. Natural drug therapy seems to be a safe and effective option for cancer patients because its main anticancer mechanism is to enhance the immune response of the host organism.

**FIGURE 1:**
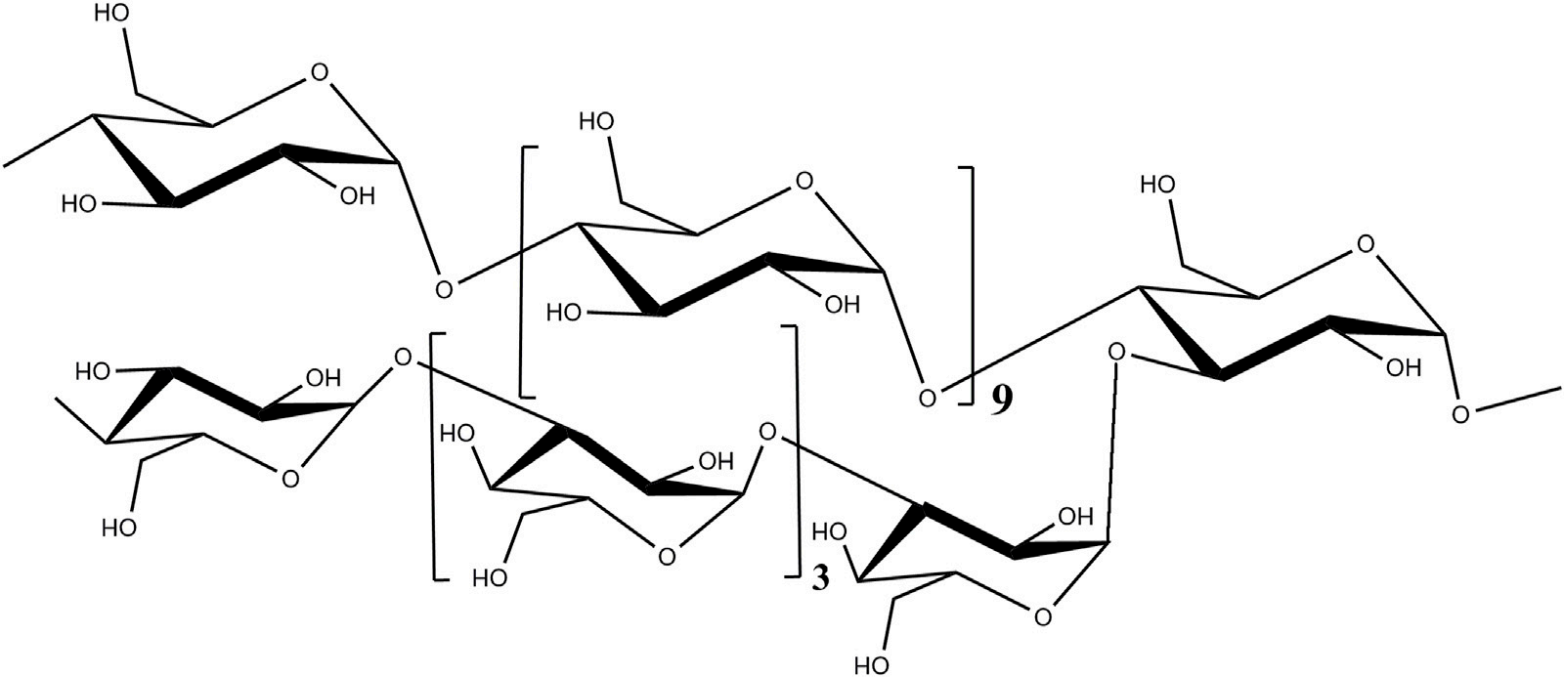
Chemical structure of SEP.

Polysaccharides are natural biological macromolecules derived from fungi, marine organisms, plants, lichens, and animals (Ooi and Liu, 2000). Previous studies have shown that polysaccharides and polysaccharide–protein complexes exhibit significant anticancer activities and increase the efficacy of other small-molecule chemotherapy drugs (Zong et al., 2012; Zhang et al., 2017; Wang M. et al., 2011; Xie et al., 2019; Wang et al., 2020). Polysaccharides are superior in inhibiting tumor metastasis. The main characteristic of polysaccharides with immune activity is the importance of their structure–function relationships (Chang, 2002). *Strongylocentrotus nudus* egg polysaccharide (SEP), a *D*-glucan containing an *α*-1, 4-linked backbone and *α*-1, 3-linked branches (structure shown in Figure 1), is extracted and purified from *Strongylocentrotus nudus* (sea urchins) eggs. This polysaccharide is widely accepted as a bioactive anticancer compound with profound inhibitory effects on tumor growth (Gao et al., 2011), which are closely related to its immunomodulatory biological activity. Numerous pharmacological studies have indicated that SEP is an anticancer candidate working through the following mechanisms: 1) promoting anti-lung cancer ability by mediating NK cytotoxicity via TLR2 and TLR4 (Ke et al., 2014); 2) preventing hepatocarcinoma by enhancing host immune system function, including by stimulating splenocyte proliferation and IL-2 and TNF secretion, as well as elevating immune globulin levels in the serum (Wang M. et al., 2011); 3) upregulating IL-2, TNF-α, and IFN-γ mRNA expression (Liu et al., 2008a); and 4) increasing mRNA expression of inducible nitric oxide synthase (iNOS) (Liu et al., 2008b). Furthermore, it has been proven that SEP can work against myelosuppression and immunosuppression in cyclophosphamide-treated mice (Wang H. et al., 2011).

As SEP is a biological macromolecule with a molecular weight of 6.78*10^5^ Da, it is difficult to determine its concentration in the biological matrix by traditional methods, such as LC-MS/MS and HPLC-UV. In a previous report, SEP was analyzed through the fluorescence labeling method (Dalvie, 2000). However, this method failed to distinguish the behavior of SEP and FITC-SEP in rats by ignoring the impact of FITC on SEP. The pharmacokinetic study of macromolecules has always been a challenge. To solve this problem, many efforts have been made in past reports, and the isotope-labeling tracer method has been proven to be a more appropriate way to study the pharmacokinetics of bio-macromolecular drugs in animals. For example, the tritium labeling method was employed by Jiang et al. (2015) to study the D-peptide D3 in mice; the ^3^H-labeling method was used to obtain the pharmacokinetic behaviors of calf thymus DNA in rats and beagles (Yang et al., 2012a; Yang et al., 2014). Compared with former methods, such as LC-MS/MS and HPLC-UV, the isotope-labeling method has the advantages of being sensitive, accurate, and conforming to physiological conditions *in vivo* (Edelmann, 2022). Thus, in the present study, SEP was radiolabeled and quantified using a sensitive liquid scintillation counter (LSC) method, and its pharmacokinetic profile in rats and beagles was investigated.

## 2 Materials and methods

### 2.1 Chemicals and reagents

Unlabeled SEP was provided by the School of Life Science and Technology, China Pharmaceutical University (Nanjing, China). Scintillation fluid was from PerkinElmer Life Science (Boston, MA). Isopropanol was purchased from Tedia (USA). Purified water was prepared using the Milli-Q water purification system (Millipore, USA). Other chemicals used in the experiment were commercially available analytical reagents.

### 2.2 Radiolabeling of SEP

Radiolabeled SEP was synthesized using an isotope exchange method. Specifically, a certain amount of SEP was dissolved in deionized water, and then 10% palladium/carbon (Pd/C) was added as catalyst. A round-bottomed flask with a magnetic stir bar was attached to a gas transfer manifold. The contents were frozen with liquid nitrogen, and tritium gas was added via standard vacuum transfer techniques. Then, the exchange reaction was conducted at 25°C with continuous stirring for 40 h. The tritium gas was finally removed, and the reaction was terminated with freezing liquid nitrogen.

### 2.3 Radiochemical purity detection

Thin-layer chromatography (TLC) was applied to analyze the radiochemical purity of ^3^H-SEP. ^3^H-SEP was dissolved in water. The sample was dotted at one corner of the thin-layer silica gel plate and developed at room temperature with methanol. After the sample had dried, the plate was pressed, exposed, and scanned on a phosphor screen imager.

### 2.4 Sample preparation

An aliquot of 50 μL rat plasma sample was transferred into a liquid flash bottle. A volume of 1 mL of scintillation cocktail was added and mixed for 1 min before analysis.

### 2.5 Method validation

#### 2.5.1 Background value and calibration curve

The detection of background value was carried out to obtain credible data by analyzing eight batches of blank plasma.

Calibration curve samples consisted of radioactive/non-radioactive SEP-mixed solution at seven radioactivity levels and blank plasma. The detection of ^3^H-SEP was treated as ordinates (Y) and the theoretical ones as abscissa (X). The range of the calibration curves for both rats and beagles was 4–400 Bq. The linearity of the method was evaluated by a least-squares linear regression to obtain calibration equations containing eight non-zero concentrations.

Limit of detection (LOD) was calculated based on background value.

#### 2.5.2 Accuracy and precision

Accuracy and precision were determined using five replicate runs at three levels of quality control (QC) and LLOQ samples on three consecutive days. Relative error (RE) and relative standard deviation (RSD) were adopted to express accuracy and precision, respectively. Percentage of accuracy within ±15%, and inter-and intra-day precision not beyond 15% were accepted for QC samples, except for the LLOQ, which should be within 20%.

#### 2.5.3 Quenching effect

The quenching effect in the ^3^H-labeled experiment was equal to the recovery or matrix effect in traditional pharmacokinetic studies. Five replicate samples, of three different levels mixed with blank plasma, were compared with three levels of working solutions. The ratio of the radioactivity of samples with blank plasma to the radioactivity of working solutions was investigated to estimate the quenching effect.

#### 2.5.4 Stability

The stability of ^3^H-SEP in plasma was estimated by analyzing five replicates in four situations: at room temperature (25°C) for 4 h, at 4°C for 24 h, at −20°C for 30 days, and in three freeze–thaw cycles from −20°C to room temperature.

### 2.6 Pharmacokinetic studies

Sprague–Dawley rats (220 ± 10 g) and beagles (11.0 ± 0.5 kg) were purchased from Shanghai Sippr-BK Laboratory Animal Co. Ltd (Shanghai, China) and Nanjing Yadong Biotechnology Co. Ltd (Nanjing, China), respectively. The rats and dogs were kept in an environment with a 12-h-on/12-h-off cycle and constant temperature and humidity. All animals were fasted for 12 h before the experiment, with free access to water. All animal studies were carried out according to standard operating procedures, and the ethical norms were approved by the Animal Ethics Committee of China Pharmaceutical University.

To achieve the desired SEP concentration, non-labeled SEP was added to radioactive working solution. The radioactivity of the animal experiment was 40 μCi/kg for rats and 6 μCi/kg for beagles. A total of 18 SD rats of each gender were divided randomly into three groups of six rats. Low (20 mg/kg, the ratio between cold and hot SEP: 319:1), middle (40 mg/kg, the ratio between cold and hot SEP: 639:1), and high (80 mg/kg, the ratio between cold and hot SEP: 1,279:1) levels of SEP and ^3^H-SEP solutions of physiological saline were administered by intravenous tail injection once per day for single-dose administration and multiple doses each day consecutively for 7 days for multiple-dose administration. Blood samples of rats were collected in heparinized tubes via the orbital venous plexus before dosing and at 0.033, 0.083, 0.25, 0.5, 1, 2, 4, 8, 12, 24, 48, 72, and 96 h after administration.

The beagle study also consisted of three groups, with four dogs in each group. The doses of SEP in beagle dogs were 5 mg/kg, 10 mg/kg, and 20 mg/kg, and the ratios of cold to hot SEP were 79:1, 159:1, and 319:1, respectively. The administration dosage was determined according to previous pharmacodynamic studies, and the experimental operation is described in the previous paragraph. Biological samples from dogs were obtained from a forelimb vein, and the sampling times were 0, 0.033, 0.083, 0.25, 0.5, 1, 2, 6, 12, 24, 48, 72, and 96 h after dosing. All samples were centrifuged immediately at 8,000 rpm for 5 min to obtain the plasma and were stored at −20°C to avoid transformation between ^3^H-labeled SEP and H_2_O.

### 2.7 Data analysis

Pharmacokinetic parameters were calculated by a non-compartmental model using Drug and Statistics (version 2.1, Mathematical Pharmacology Professional Committee of China, Shanghai, China). Data were expressed as mean values ± standard deviation (mean ± SD).

## 3 Results

### 3.1 Radioactivity verification of ^3^H-labeled SEP

In the present study, isotope exchange was applied to synthesize ^3^H-labeled SEP. According to the data of phosphorimager scans, the radioactivity of ^3^H-labeled SEP determined by TLC was more than 97%, which is sufficient to study the pharmacokinetics of ^3^H-SEP in rats and beagles. Analysis of diluted ^3^H-SEP from the initial radioactive products produced three measurements that demonstrated the total radioactivity of ^3^H-SEP to be 2.8 mCi. The specific activity of final preparation, as measured using the refractive index detector and liquid scintillation counter, was 0.64 mCi/mg.

### 3.2 Method validation

As a kind of biological macromolecule, polysaccharides require special consideration in the choice of research methods. Compared with other analytical methods, the isotope tracer method was selected for its high sensitivity and because it causes no changes in the properties of the drug itself. The whole process of sample determination is convenient, simple, and fast. The LSC method used to determine ^3^H-SEP in plasma was fully validated for calibration, accuracy, precision, stability, and LLOQ according to the US FDA guidelines (U.S. Food and Drug Administration Bioanalytical Method Validation Guidance for Industry [2018]), followed by further instructions, including background, quenching effect, and tritium–water exchange rate.

#### 3.2.1 Background value and linearity of the calibration curve

The background value of LSC is important to ^3^H-labeled SEP and is closely related to the sensitivity of detection expressed by LLOQ. Before each sample measurement, background values should be verified to ensure that the instrument has not been contaminated. In this test, the LLOQ of ^3^H-SEP was 4 Bq, which ensured that the analysis of experiment samples was accurate. The correlation coefficient (r) of standard calibration curves was 1, showing good linearity. The regression equation of the calibration curve was Y = 0.333 + 0.980X. The limit of detection (LOD) was 1 Bq.

#### 3.2.2 Accuracy and precision

The intra-day and inter-day precision of ^3^H-SEP in blank plasma of rats and beagles were less than 3.0% and 3.9%, respectively, of all levels (8 Bq, 40 Bq, and 320 Bq). As shown in Table 1, the accuracy ranged from 95.0% to 102.1%. These results show that the LSC method is very stable, which is also one of the advantages of isotope tracer methods. It is also considered to be reasonable and reliable to analyze the ^3^H-SEP radioactivity in biological matrices of rats or dogs.

**TABLE 1:** Precision and accuracy results for the radioactivity determination of ^3^H-SEP in rat and beagle plasma.

Radiation volume (Bq)	Intra-day (*n* = 5)		Inter-day (*n* = 15)	
	Accuracy (mean ± SD, %)	Precision (RSD, %)	Accuracy (mean ± SD, %)	Precision (RSD, %)
Rats				
4	99.8 ± 2.3	6.8	98.9 ± 1.8	1.7
8	98.9 ± 3.9	3.0	102.7 ± 3.5	3.9
40	99.2 ± 3.5	2.1	100.4 ± 2.0	3.0
320	98.6 ± 1.5	1.1	96.7 ± 1.1	1.9
Beagles				
4	97.5 ± 1.3	7.6	98.5 ± 1.5	1.8
8	92.9 ± 3.0	3.2	93.3 ± 3.3	3.5
40	100.3 ± 1.8	1.8	98.2 ± 2.5	1.9
320	99.3 ± 1.6	1.6	99.7 ± 1.6	1.6

#### 3.2.3 Quenching effect

The quenching effect is the exclusive characteristic of the determination of LSC, which demonstrates the influence of plasma on radioactivity analysis. In the current study, the blank rat or dog plasma mixed with stock solution had no obvious effect on the analysis. In the protocol, the results suggest that no serious quenching effect existed in biological matrices.

#### 3.2.4 Stability

The tritium–water exchange rate is another way to verify the stability of ^3^H-SEP in diluent and plasma, either in rats or in beagles. As shown in Table 2, there was no significant change in the stability of QC samples under different conditions. The deviation was within 8.2% of theoretical concentration under four established conditions.

**TABLE 2:** Stability results for ^3^H-SEP in rat and beagle plasma.

Stability condition	Radiation volume (Bq)	Mean ± SD	RSD (%)
Rat			
Short-term stability (25°C, 4 h)	8	7.9 ± 0.4	4.5
	40	40.0 ± 1.3	3.3
	320	314.9 ± 2.1	0.7
Post-preparative stability (4°C, 24 h)	8	8.3 ± 0.7	8.2
	40	37.9 ± 0.7	2.0
	320	316.5 ± 2.5	0.8
Long-term storage stability (-20°C, 30 days)	8	8.0 ± 0.2	2.6
	40	39.5 ± 0.7	1.8
	320	311.4 ± 3.4	1.1
Three freeze–thaw cycles (-20°C)	8	8.0 ± 0.3	3.5
	40	39.1 ± 1.4	3.5
	320	305.9 ± 2.7	0.9
Beagle			
Short-term stability (25°C, 4 h)	8	7.3 ± 0.2	3.2
	40	39.8 ± 0.7	1.6
	320	315.8 ± 2.9	0.9
Post-preparative stability (4°C, 24 h)	8	7.51 ± 0.2	2.6
	40	39.7 ± 0.4	0.9
	320	318.9 ± 5.4	1.7
Long-term storage stability (−20°C, 30 days)	8	7.4 ± 0.3	4.4
	40	39.8 ± 0.5	1.4
	320	316.4 ± 1.9	0.6
Three freeze–thaw cycles (-20°C)	8	7.2 ± 0.1	1.1
	40	39.9 ± 0.6	1.4
	320	321.7 ± 2.3	0.7

### 3.3 Pharmacokinetic study

**FIGURE 2:**
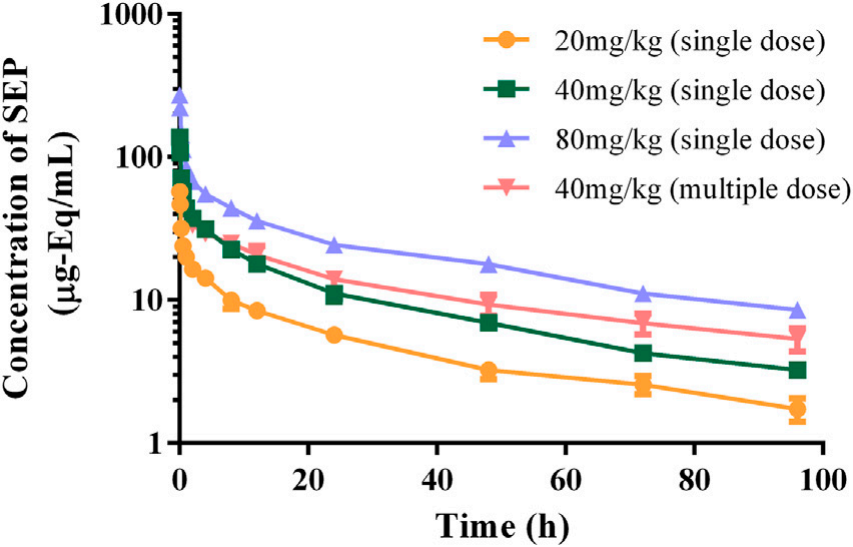
Logarithmic graph of mean plasma concentration–time profiles in rats after intravenous injection of SEP (single doses of 20, 40, or 80 mg/kg or multiple doses of 40 mg/kg on the seventh day).

**FIGURE 3:**
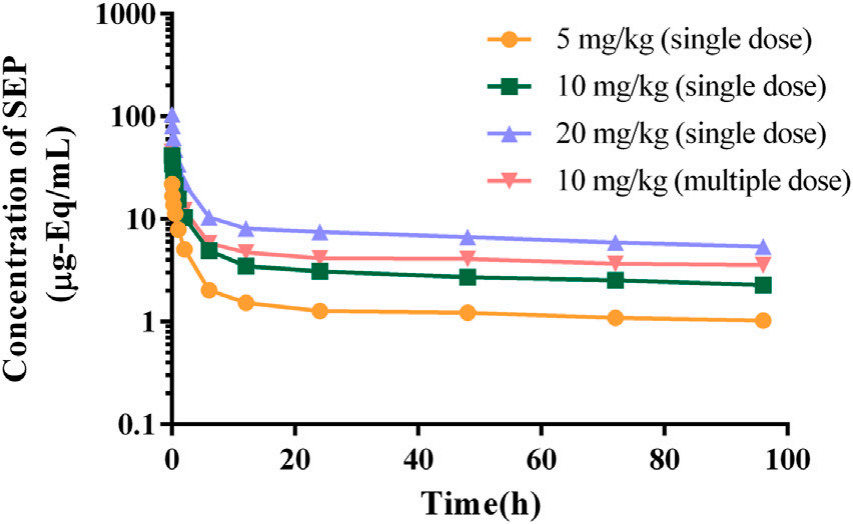
Logarithmic graph of mean plasma concentration–time profiles in beagles after intravenous injection of SEP (single doses of 5, 10, or 20 mg/kg or multiple doses of 10 mg/kg on the seventh day).

The authenticity value of SEP was calculated based on ^3^H-SEP radioactivity in rat and beagle plasma after intravenous administration. It was sensitive enough to determine the plasma concentration up to 96.0 h. The mean plasma concentration–time profiles of SEP are shown in Figures 2, 3, and calculated pharmacokinetic parameters are exhibited in Table 3. The concentration of SEP in plasma from different species and three dose groups declined with a similar trend, dramatically decreasing within 30 min of administration and being eliminated slowly. Compared with single administration, concentrations determined at each point for multiple administration were all relatively high. The half-life of SEP is 35.48 and 204.29 h in rats and dogs, respectively. As shown in Table 3, the AUC_(0-t)_ value of SEP increased proportionately with dose in both rats and beagles. The CL_z_ values for the middle- and high-dose-level groups were slightly higher than that of the low-level dose group.

**TABLE 3:** Pharmacokinetic parameters of SEP after intravenous injection in rats (*n* = 6) and beagles (*n* = 4).

Parameter	Rat			
	20 mg/kg	40 mg/kg	80 mg/kg	40 mg/kg (multiple dose)
AUC_(0-t)__(_mg/L*h)	487.81 ± 39.99	1,003.10 ± 95.94	2,188.84 ± 137.73	1,216.00 ± 134.12
AUC_(0-∞)__(_mg/L*h)	599.91 ± 65.65	1,148.35 ± 111.49	2,772.41 ± 261.40	1,656.72 ± 235.18
t_1/2z_ (h)	43.33 ± 6.11	35.48 ± 6.04	46.51 ± 8.46	55.93 ± 17.41
T_max_ (h)	0.03 (0.03,0.03)	0.03 (0.03,0.03)	0.03 (0.03,0.03)	0.03 (0.03,0.03)
CL_z_ (L/h/kg)	0.03 ± 0.00	0.04 ± 0.00	0.03 ± 0.00	0.04 ± 0.00
V_z_ (L/kg)	2.09 ± 0.21	1.79 ± 0.31	1.93 ± 0.21	1.90 ± 0.57
C_max_ (mg/L)	58.69 ± 4.86	140.72 ± 7.86	277.96 ± 13.90	136.95 ± 9.24

Parameter	Beagle			
	5 mg/kg	10 mg/kg	20 mg/kg	10 mg/kg (multiple dose)
Parameter	5 mg/kg	10 mg/kg	20 mg/kg	10 mg/kg (multiple dose)
AUC_(0-t)__(_mg/L*h)	144.12 ± 3.78	322.62 ± 28.03	754.17 ± 37.79	517.76 ± 28.34
AUC_(0-∞)__(_mg/L*h)	474.45 ± 205.71	1,051.64 ± 648.17	1941.52 ± 158.62	1831.56 ± 461.00
t_1/2z_ (h)	221.61 ± 121.30	204.29 ± 139.34	153.88 ± 24.15	279.45 ± 461.00
T_max_ (h)	0.03 (0.03,0.03)	0.03 (0.03,0.03)	0.03 (0.03,0.03)	0.03 (0.03,0.03)
CL_z_ (L/h/kg)	0.01 ± 0.01	0.01 ± 0.01	0.03 ± 0.00	0.01 ± 0.00
V_z_ (L/kg)	3.23 ± 0.50	2.74 ± 0.17	2.28 ± 0.18	2.85 ± 0.31
C_max_ (mg/L)	22.03 ± 0.92	42.00 ± 5.42	105.19 ± 10.81	45.35 ± 1.36

Note: All data are presented as mean ± SD, except for T_max_ values, presented as median (min, max).

## 4 Discussion

Marine anticancer drug research has always played a leading role in marine drug research (Khalifa et al., 2019; Ren et al., 2021). Compared with chemical synthetic drugs, pharmaceutical ingredients taken from natural marine organisms have lower toxicity and higher safety, and they can selectively identify cancer-related factors, thereby inhibiting the growth or causing the death of tumor cells (de Jesus Raposo et al., 2015). At the same time, these ingredients avoid damage to normal cells in the human body, effectively treat the disease, and greatly reduce the side effects of drugs. SEP extracted from *Strongylocentrotus nudus* sea urchin eggs has the advantage of natural anticancer active ingredients. Unlike synthetic chemical compounds, SEP is a biological macromolecule with a molecular weight of 1.63*10^5^ Da, so it is hard to identify it quantitatively and to conduct pharmacokinetic studies for SEP using conventional methods. Radioisotopes have been proven to be essential in biomedical study and have played a vital role in studying the pharmacokinetic properties of new chemical compounds, including their absorption, distribution, metabolism, and excretion (ADME) (Dalvie, 2000). Common methods of isotope labeling include chemical synthesis, biochemistry, and isotope exchange, and the most widely used nuclides include 3H, 14C, 35S, 32P, 76Br, and 125I (Fu et al., 2010). In this study, tritium was selected as the tracer to label SEP to investigate the pharmacokinetics of SEP in rats and beagles.

According to the results of method verification, the tritium tracer method is stable, guaranteeing the authenticity and reliability of the SEP pharmacokinetic results. In rats and beagles, the concentration of SEP in plasma was reduced by 90% within 24 h of administration, while the remaining 10% was metabolized more slowly. This phenomenon may be related to the characteristics of tritium-labeled isotope tracing (Yang et al., 2012b). In this study, radiation rather than SEP was quantitatively measured, and the concentration of SEP was then calculated based on the proportion. Therefore, the radiation measured after 24 h is likely not derived from ^3^H-SEP but from SEP metabolites or metabolized tritium water (Yang et al., 2012b). It is worth noting that its pharmacokinetic profile does not meet the typical compartment model, possibly because the plasma concentration determined was converted from the total radioactivity, which was the sum of parent SEP and its radioactive metabolites; this is a shortcoming of the tritium tracer method. This is also why the AUC extrapolation ratio of beagles is too large. Compared to AUC_(0-∞)_, the AUC_(0-t)_ of SEP is more credible and should be used as a basis for further development.

Being similar to traditional chemicals, multiple dosing administration of SEP was also applied to investigate the possibility of accumulation. In general, anticancer drugs must be administered continuously, so the accumulated results in the body should be noted in future clinical studies. The results of this study preliminarily show the application of tritium marker tracer SEP in rats and beagles, providing a new idea for the study of polysaccharide pharmacokinetics and forming the basis of further research on the pharmacokinetics and pharmacodynamics of SEP. In other words, the relationship between the concentration of SEP at the target site and the efficacy concentration requires more attention.

## 5 Conclusion

Tritium-labeled SEP was synthesized, and a reliable LSC radioisotope detection method was developed and successfully applied to pharmacokinetic studies in rats and beagles. An apparent decrease was observed after intravenous injection, according to the plasma concentration results, which was contrary to elimination in both rats and beagles. This study further investigated the pharmacokinetics of SEP *in vivo* and will promote the development of SEP as a new anticancer candidate.

## Data Availability

The raw data supporting the conclusion of this article will be made available by the authors, without undue reservation.
